# Airborne PM_2.5_-Induced Hepatic Insulin Resistance by Nrf2/JNK-Mediated Signaling Pathway

**DOI:** 10.3390/ijerph14070787

**Published:** 2017-07-14

**Authors:** Jinxia Xu, Wei Zhang, Zhongbing Lu, Fang Zhang, Wenjun Ding

**Affiliations:** 1 Laboratory of Environment and Health, College of Life Sciences, University of Chinese Academy of Sciences, No. 19A Yuquan Road, Beijing 100049, China; xujinxia13@mails.ucas.ac.cn (J.X.); zhangw@ucas.ac.cn (W.Z.); zhangfang@ucas.ac.cn (F.Z.); 2 Sino-Danish College, University of Chinese Academy of Sciences, No. 3 Zhongguancun South 1st Alley, Beijing 100190, China

**Keywords:** PM_2.5_, liver, insulin resistance, oxidative stress, Nrf2, JNK/IRS-1/AKT

## Abstract

Animal and epidemiological studies have suggested that exposure to airborne particulate matter (PM) with an aerodynamic diameter less than 2.5 μm (PM_2.5_) is associated with the risk of developing type 2 diabetes. However, the mechanism underlying this risk is poorly understood. In the present study, we investigated the effects of PM_2.5_ exposure on glucose homeostasis and related signaling pathways in mice. Wild-type and nuclear factor erythroid 2-related factor 2 (Nrf2) knockout (Nrf2^−/−^) C57BL/6 male mice were exposed to either ambient concentrated PM_2.5_ or filtered air (FA) for 12 weeks through a whole-body PM exposure system. At the end of the exposure, we assessed liver damage, and performed metabolic studies, gene expressions, as well as molecular signal transductions to determine the signaling pathways involving oxidative responses, insulin signaling, and glucose metabolism. Our results indicated that PM_2.5_ exposure for 12 weeks caused significant liver damage as evidenced by elevated levels of aminotransferase (AST) and alanine aminotransferase (ALT). Furthermore, PM_2.5_ exposure induced impaired glucose tolerance and inhibited glycogen synthesis, leading to hepatic insulin resistance indicated by higher glucose levels, higher area under the curve (AUC), and homeostasis model assessment of insulin resistance (HOMA-IR) values. We further found that PM_2.5_ exposure significantly increased the expressions of Nrf2 and Nrf2-regulated antioxidant genes. Moreover, PM_2.5_ exposure activated the c-Jun N-terminal kinase (JNK) signaling pathway and increased insulin receptor substrate-1 (IRS-1) phosphorylation at Ser^307^, but reduced protein kinase B phosphorylation at Ser^473^. Taken together, our study demonstrated PM_2.5_ exposure triggered Nrf2-mediated oxidative responses and activated the JNK-mediated inhibitory signaling pathway, resulting in hepatic insulin resistance.

## 1. Introduction

Some of the health risks of exposure to airborne particulate matter (PM) with an aerodynamic diameter less than 2.5 μm (PM_2.5_), such as the impacts on the respiratory and cardiovascular systems, have been extensively studied [1,2]. These fine particles enter the body easily, deposit in the lung, and even enter into the circulatory system, resulting in health risks through different pathological processes [3,4,5]. Recently, numerous epidemiological studies have also revealed that exposure to air pollution may be associated with an increased risk for developing diabetes mellitus (DM) [6,7,8,9,10,11].

Insulin resistance usually refers to a defect in the ability of insulin to stimulate glucose uptake and is a characteristic feature of DM, obesity [12], and other metabolic diseases [13]. It is noteworthy that IR is characterized by impairment of the insulin-induced activation of the insulin receptor substrate (IRS)/phosphoinositide 3-kinase (PI3K)/protein kinase B (AKT) pathway, leading to suppression of the insulin-induced glucose uptake in the insulin-sensitive organs, such as the liver [14].

PM_2.5_-induced oxidative stress has been considered as a key molecular mechanism of PM_2.5_-mediated toxicity [15,16]. Emerging evidence has suggested that oxidative stress plays a causal role in the complications of insulin resistance (IR), and over-generation of reactive oxygen species (ROS) and insulin resistance may be co-conspirators in liver dysfunction, each capable of triggering or worsening the other [17,18]. In addition, recent studies have showed that PM_2.5_-triggered systemic and pulmonary inflammation induce a non-alcoholic steatohepatitis (NASH)-like phenotype and impair hepatic glucose metabolism in an animal model [19,20]. However, a direct relationship between PM_2.5_-induced oxidative stress and hepatic insulin resistance has not been established.

Redox-sensitive nuclear factor erythroid 2-related factor 2 (Nrf2) is a key regulatory transcription factor which regulates antioxidant response element (ARE)-mediated expression of detoxifying and antioxidant enzymes that protect against the adverse effects of oxidative stress induced by ROS [21,22]. It has been demonstrated that diminished Nrf2/ARE activity contributes to oxidative stress, leading to endothelial dysfunction and insulin resistance in diabetes [23,24]. Regarding with cytoprotection, involvement of Nrf2 in diabetes mellitus and obesity has been suggested. For example, streptozotocin-induced diabetes in Nrf2-null mice exhibits increases in oxidative and nitrosative stress levels [25], as well as elevated blood glucose levels, via enhanced expression of hepatic gluconeogenesis-related genes [26]. Therefore, we conceived that Nrf2 is involved in glucose homeostasis and is a crucial player in the regulation of insulin signaling in the liver.

Previous studies have showed that PM_2.5_ exaggerates IR in mice fed either a high-fat diet or a normal diet [18,20,27,28]. The insulin receptor substrate-1 (IRS-1)/protein kinase B (AKT) signal pathway is a crucial classical insulin signal pathway in glucose metabolism [29]. Glucose and insulin homeostasis are disrupted when the IRS-1/AKT signal pathway is suppressed through increasing phosphorylation of IRS-1 at serine residues, as well as decreasing the expression levels of IRS-1/AKT [29,30,31,32,33,34]. In addition, the activated PI3K/AKT signal pathway could accelerate redox-sensitive nuclear factor Nrf2 translocation [35,36]. Nrf2 is essential for the coordinate induction of phase II detoxifying enzymes and is a regulator of the defense genes against oxidative stress [37]. It has been reported that PM_2.5_ mediates IR by regulating hepatic lipid metabolism, and glucose utilization in skeletal muscle [28,31]. In the present study, we used a whole-body exposure model of mice to investigate the effects of PM_2.5_ exposure on glycometabolism and explore the signaling pathways involved in oxidative stress and insulin sensitivity.

Long-term ambient PM_2.5_ exposure has been reported to induce impaired glucose tolerance, IR, inflammation and mitochondrial alteration in adipose tissue [28]. Since liver plays a key role in maintaining blood glucose homeostasis, in this study, we focused on the impacts of PM_2.5_ exposure on the liver to determine the effects of PM_2.5_ exposure on glucose homeostasis, and explore the molecular signaling pathways associated with glucose metabolism.

## 2. Materials and Methods

### 2.1. Reagents and Antibodies

Insulin reagents kit was purchased from Amresco (Solon, OH, USA). The commercial kits for quantifying hepatic glycogen, alanine aminotransferase (ALT), aspartate aminotransferase (AST), superoxide dismutase (SOD), catalase (CAT), glutathione (GSH), and malonaldehyde (MDA) were purchased from Nanjing Jiancheng Bioengineering Institute (Nanjing, China). Mouse 8-hydroxy-desoxyguanosine (8-OHdG) ELISA kit was purchased from HongYueChuanXin Biotech Co., Ltd. (Beijing, China). The enzyme-linked immunosorbent assay kit for tumor necrosis factor-α (TNF-α) was purchased from PeproTech, Inc. (Rocky Hill, NJ, USA). Trizol agent was purchased from Invitrogen (Carlsbad, CA, USA). RNA reverse transcription reagents were from Promega (Madison, WI, USA). UltraSYBR mixture and β-actin antibody were purchased from Beijing CoWin Bioscience (Beijing, China). Protease inhibitor cocktail was purchased from Selleck Chemicals (Houston, TX, USA). Phosphatase inhibitor was purchased from Roche (Basel, Switzerland). BCA protein assay kit was purchased from Tiangen Biotech Co., Ltd. (Beijing, China). Electrochemiluminescence (ECL) reagents were purchased from BIO-RAD (Hercules, CA, USA). Antibodies against phosphorylated JNK (Thr183/Tyr185) (1:1000) and total JNK (1:1000) were purchased from Santa Cruz Biotechnologies, Inc. (Santa Cruz, CA, USA). Antibodies against heme oxygenase-1 (HO-1) (1:1000), phosphorylated IRS-1 (Ser^307^) (1:500), total IRS-1 (1:500), phosphorylated AKT (Ser^473^) (1:1000) and total AKT (1:1000) were purchased from Cell Signaling Technologies (Danvers, IL, USA). Radio immunoprecipitation assay lysis buffer (RIPA), HRP-labeled goat anti-rabbit IgG (1:8000) and HRP-labeled goat anti-mouse IgG (1:1000) were purchased from Beyotime (Haimen, China). All other chemicals used were of analytical grade.

### 2.2. Animals

Five-week-old male C57BL/6 wild-type (WT) mice were purchased from the Experimental Animal Center, the First Hospital Affiliated to Chinese People’s Liberation Army General Hospital. Male Nrf2^−/−^ (Nrf2) mice were purchased from Jackson Laboratory (Bar Harbor, ME, USA). All experiments and protocols described here were approved by Experimental Animal Centre, the First Hospital Affiliated to Chinese People’s Liberation Army General Hospital. The animals were maintained in a pathogen-free animal facility with a 12 h light/dark cycle (24 °C) and had free access to water and standard laboratory chow. All mice were cared in accordance with ethical guidelines set forth by the College of Life Sciences of University of Chinese of Academy of Sciences (UCAS), with Institutional Animal Care and Use Committee (IACUC) #04-2016-01.

### 2.3. Experimental Design

Both WT and Nrf2 mice were randomly assigned to two groups, respectively. Animals were exposed to either ambient PM_2.5_ or filtered air (FA) for 12 h/day, five days/week, for 12 weeks (May–August, 2015) in a “real-world” airborne PM exposure system in ZhongGuanCun Campus of the University of Chinese Academy of Sciences [38]. The animal groups were as follows: WT-FA (*n* = 6), WT-PM_2.5_ (*n* = 6), Nrf2-FA (*n* = 6), and Nrf2-PM_2.5_ (*n* = 6). The mice in the device were fed commercial mouse chow and distilled water and were housed under controlled temperature (22 ± 2 °C) and relative humidity (40–60%) conditions with a 12 h light/dark cycle. During the exposure time period, the mean daily ambient PM_2.5_ concentration at the study site was 64 μg/m^3^ (the annual average PM_2.5_ National Ambient Air Quality Standard (NAAQS) of 15 μg/m^3^ in China). The body weight and the levels of fasted blood glucose were measured every week.

### 2.4. Blood and Tissue Collection and Homogenate Preparation

After 12-week exposure, the mice were anesthetized with ether. Blood was collected from the abdominal vein, and separated at 3000 rpm for 10 min to obtain serum. Serum was stored at −80 °C for the analysis the levels of ALT, AST, and insulin. The livers were perfused in situ with saline and were then immediately removed. Then the tissue was stored at −80 °C. Liver was homogenized in sterile saline using an electric homogenizer, then centrifuged at 3500 rpm for 15 min [39]. The supernatants were stored at −80 °C for analysis of antioxidant enzymes activity.

### 2.5. Measurement of Glucose and Hormone Levels

Two days before the end of exposure, oral glucose tolerance tests (OGTT) were performed by glucose administration (2 mg/g) to WT mice and Nrf2^−/−^ mice fasted 15 h [40]. Blood glucose was determined by measuring tail blood concentrations at 0, 30, 60 and 120 min after glucose administration, respectively. The area under the curve (AUC) was calculated with the following formula:

(1) AUC=0.25×(B0)+0.5×(B30)+0.75×(B60)+0.5×(B120)

B0, B30, B60, and B120 mean the values of glucose in OGTT. The serum levels of insulin were also determined by radioimmunaossay using reagents kits. Homeostasis model assessment of insulin resistance (HOMA-IR) was calculated by the following formula:

(2) $$\text{HOMA-IR}=\frac{\mathrm{FINS}(\mu U/\mathrm{mL})\;\times \;\mathrm{FBG}\;(\mathrm{mmol}/L)}{22.5}$$

where FINS means fasting insulin and FBG means fasting blood glucose [41].

### 2.6. Biochemical Analysis

The activities of ALT and AST in serum, the activities of SOD and CAT in liver, and the levels of GSH, MDA, 8-OHdG, and glycogen in liver and TNF-α in serum were determined by a commercial assay kit according to the manufacture’s instruction, respectively.

### 2.7. Quantitative Analysis of Gene Expression

Total RNA was extracted from frozen liver using Trizol agent according to manufacturer’s instructions. The concentrations of RNA were measured with Nanodrop 2000 (Thermo Fisher, Waltham, MA, USA). Then 2 μg of total RNA was subjected to transcribe the cDNA. Real-time polymerase chain reaction (PCR) was performed with a light cycler instrument (BIO-RAD, Hercules, CA, USA) to analyze the mRNA expression levels of *Nrf2, HO-1, glycogen synthase (GS), glycogen synthase kinase 3β (GSK-3β), glucokinase (GK)* and SYBR green was used to detect the amplified products. The PCR cycle was as follows: initial denaturation at 95 °C for 30 s, followed by 40 cycles of denaturation at 95 °C for 5 s, annealing at 60 °C for 10 s and extension at 72 °C for 15 s. The primers sequences for target genes were listed in Table 1. *β*-actin was amplified to normalize the quantification results of target gene expression using the 2^−ΔCt^ method.

### 2.8. Western Blotting

Total protein was extracted from frozen liver using radio immunoprecipitation assay lysis buffer (RIPA) containing protease inhibitor cocktail (Selleck, Houston, TX, USA) and phosphatase inhibitors (Roche, Switzerland). Then the lysates were centrifuged at 14,000 rpm for 5 min at 4 °C. Supernatants were collected and protein content was determined with BCA protein assay kit. 60 μg protein was subjected to sodium dodecyl sulfate polyacrylaminde gel electrophoresis (SDS-PAGE) for 2 h. Then the gel was transferred to polyvinylidene fluoride (PVDF) membrane (Millipore, Billerica, MA, USA). The PVDF membrane was blocked with 5% non-fat milk at room temperature for 1 h. After the blocking step, the membrane was washed for three cycles of five minutes each and then incubated with the primary antibody at 4 °C overnight on a table concentrator. Before incubation with the secondary antibody for 1 h, the membrane was washed with TBST at room temperature. Protein bands on the membrane were detected with ECL reagents according to the manufacturer’s instructions using automatic chemiluminescence image analysis system (Tanon 5200, Shanghai, China). The bands of protein were analyzed using Image J 1.50 software (NIH, Bethesda, MD, USA). β-actin was used as the loading controls for total protein content.

### 2.9. Statistical Analysis

The results are expressed as the mean ± standard error (SE). The significance of differences in the study parameters among groups was determined by two-way analysis of variance (ANOVA) with a post hoc test. All statistical analyses were performed using StatView (SAS Institute Inc., Hong Kong, China). The significance of differences between two groups was determined by Student’s *t*-test. A value of *p* < 0.05 was considered to indicate statistical significance.

## 3. Results

### 3.1. Effects of PM_2.5_ Exposure on Body Weight, Consumption of Food and Water, Liver Weight, and Biochemicals in Serum

At the end of exposure, body weight, liver weight, food intake, water intake, and fasting glucose were recorded. As shown in Table 2, there was no significant difference for body weight among the four groups. However, PM_2.5_ exposure increased liver weight in WT mice. In addition, the levels of fasting glucose and insulin in serum were significantly increased in Nrf2^−/−^ mice after PM_2.5_ exposure (*p* < 0.05). We also found that the levels of serum TNF-α were increased on the fourth week, but no significant changes on the 12th week (Data not shown).

### 3.2. PM_2.5_ Exposure Induced Liver Injury

**Figure 1:**
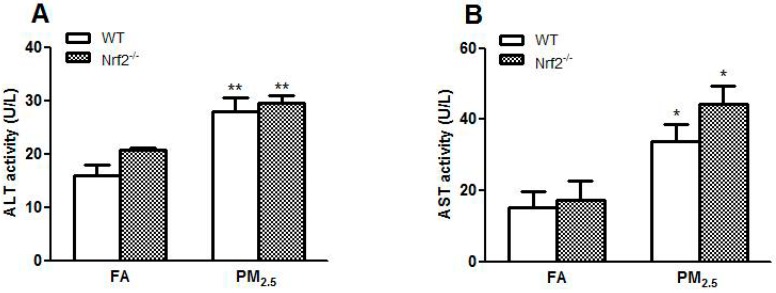
Effect of PM_2.5_ exposure on liver function in WT and Nrf2^−/−^ mice. Animals were exposed to ambient PM_2.5_ or filtered air (FA) for 12 weeks. (**A**) Serum levels of alanine aminotransferase (ALT); (**B**) serum levels of aspartate aminotransferase (AST). The results are presented as the mean ± SE (*n* = 6). * *p* < 0.05, ** *p* < 0.01 vs. the FA groups.

ALT and AST are the conventional indicators of liver damage [42]. PM_2.5_ exposure increased the serum levels of ALT (*p* < 0.01) and AST (*p* < 0.05) in the exposed mice compared to those in FA groups (Figure 1A,B), particularly in Nrf2^−/−^ mice, indicating that PM_2.5_ exposure significantly causes liver damage.

### 3.3. PM_2.5_ Exposure Induced Impaired Glucose Tolerance, and Insulin Resistance

**Figure 2:**
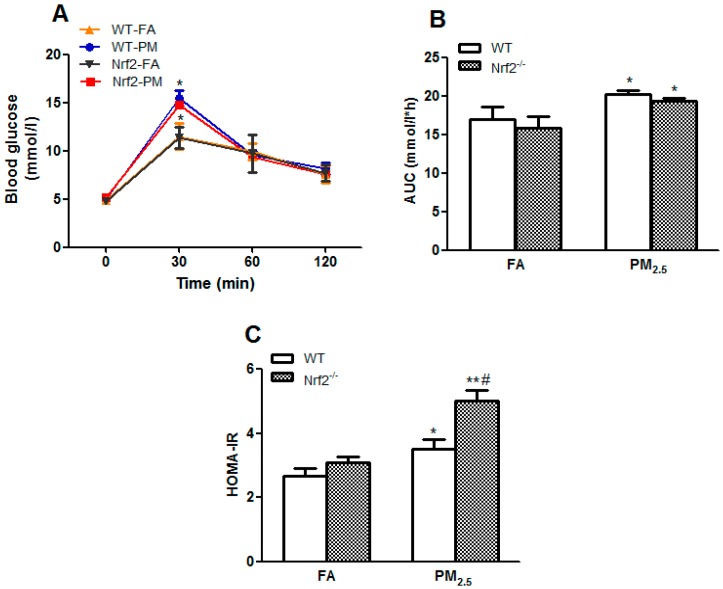
Effects of PM_2.5_ exposure on glucose homeostasis in WT and Nrf2^−/−^ mice. Animals were exposed to ambient PM_2.5_ or filtered air (FA) for 12 weeks. (**A**) Oral glucose tolerance test (OGTT); (**B**) The area under the curve (AUC); (**C**) Homeostasis model assessment of insulin resistance (HOMA-IR). The results are presented as the mean ± SE (*n* = 6). * *p* < 0.05, ** *p* < 0.01 vs. the FA groups. ^#^ *p* < 0.05 vs. the WT mice.

To assess the impacts of PM_2.5_ exposure on glucose metabolism, oral glucose tolerance test was performed. As displayed in Figure 2A, mice exposed to PM_2.5_ for 12 weeks showed significant elevations in glucose levels after oral administration of glucose for 30 min compared to the FA group, indicating that PM_2.5_ exposure induced impaired glucose tolerance in both groups of WT and Nrf2^−/−^ mice (Figure 2A). In addition, the AUC value of the PM-exposed mice was also significantly higher than that of the non-exposed mice (*p* < 0.05) (Figure 2B).

Furthermore, to evaluate IR and HOMA-IR, the indicators of insulin sensitivity [20], were calculated as described above. The results showed that the HOMA-IR value of the exposed mice was significantly higher than that of the non-exposed mice after PM_2.5_ exposure (*p* < 0.05) (Figure 2C), particularly in Nrf2^−/−^ mice, indicating that PM_2.5_ exposure induced IR.

### 3.4. PM_2.5_ Exposure Induced Impaired Glycogen Storage through Decreasing Glycogen Synthesis in Liver of Mice

**Figure 3:**
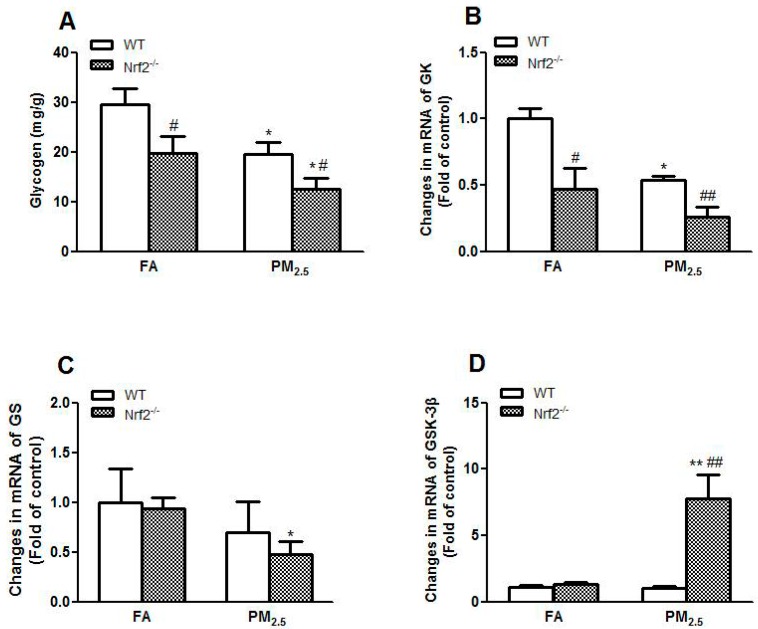
Effects of PM_2.5_ exposure on glycogen synthesis in liver of WT and Nrf2^−/−^ mice. Animals were exposed to ambient PM_2.5_ or filtered air (FA) for 12 weeks. (**A**) Hepatic glycogen. The mRNA expression levels of GK (**B**); GS (**C**); and GSK-3β (**D**) in the liver of mice after 12 weeks of exposure. The results are presented as the mean ± SE (*n* = 6). * *p* < 0.05, ** *p* < 0.01 vs. the FA groups. ^#^ *p* < 0.05, ^##^ *p* < 0.01 vs. the WT mice.

We found that Nrf2 deletion reduced the contents of hepatic glycogen (*p* < 0.05) and PM_2.5_ exposure could further aggravate this situation (*p* < 0.05) (Figure 3A). Glucokinase (GK) is an essential catalytic enzyme in the process of glycogen synthesis reaction. Nrf2 deletion decreased GK mRNA levels and PM_2.5_ exposure further inhibited GK gene expression in mice liver (Figure 3B). The mRNA expression levels of glycogen synthase (GS), which was the rate-limiting enzyme of glycogen synthesis, were obviously inhibited in Nrf2^−/−^ mice after PM_2.5_ exposure (*p* < 0.05) (Figure 3C). Meanwhile, PM_2.5_ exposure increased the mRNA expression levels of glycogen synthase kinase 3β (GSK-3β) significantly in Nrf2^−/−^ mice (*p* < 0.01) (Figure 3D). The results suggested that PM_2.5_ exposure upregulating the mRNA expression of GSK-3β decreased the content of hepatic glycogen. Meanwhile, the effects of Nrf2 deficiency on this situation were further aggravated.

### 3.5. PM_2.5_ Exposure Induced Oxidative Responses in the Liver

**Figure 4:**
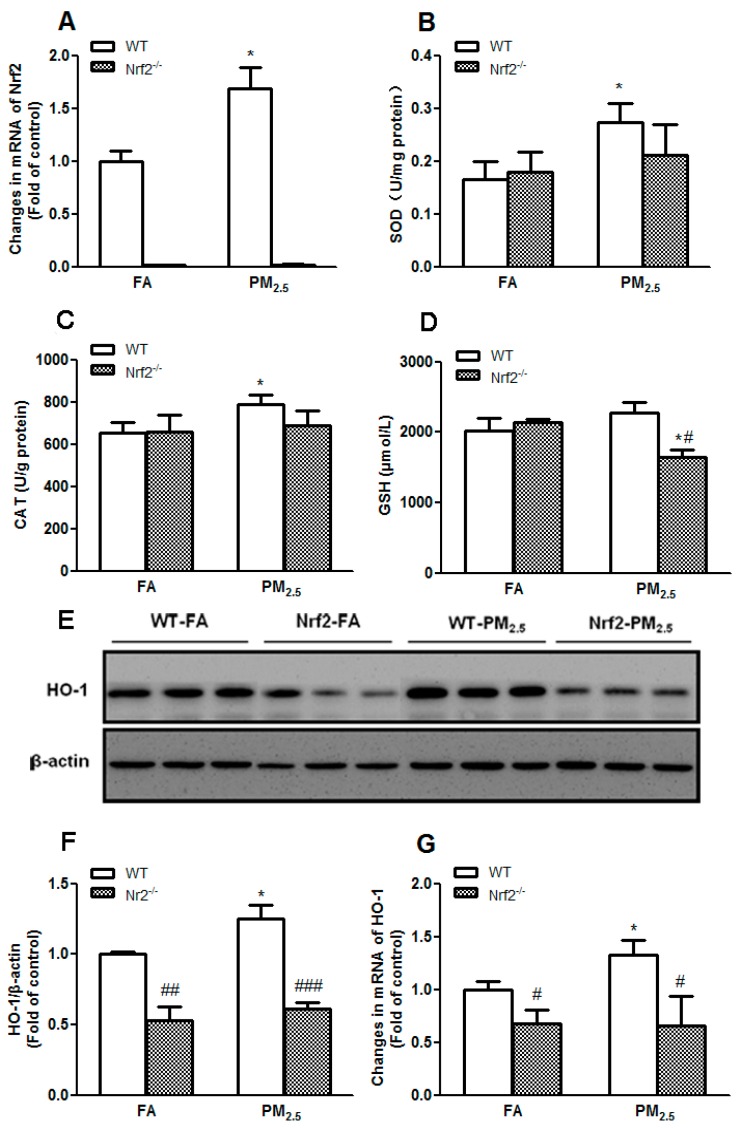
Effects of PM_2.5_ exposure on oxidative responses in the liver of WT and Nrf2^−/−^ mice. Animals were exposed to ambient PM_2.5_ or filtered air (FA) for 12 weeks. (**A**) The mRNA expression levels of Nrf2 in the liver of mice. The activities of superoxide dismutase (SOD) (**B**), catalase (CAT) (**C**), and the levels of glutathione (GSH) (**D**) in livers of the mice. The results are presented as the mean ± SE (*n* = 6). * *p* < 0.05 vs. the FA groups. ^#^ *p* < 0.05 vs. the WT mice. Representative Western blots (**E**) and quantitative data of HO-1 (**F**) in liver of mice are shown. β-actin was blotted as a loading control; (**G**) the mRNA expression levels of HO-1 in the liver of mice exposed to PM_2.5_ or filtered air (FA) for 12 weeks. All values given are the mean ± SE of three independent experiments. * *p* < 0.05 vs. the FA groups. ^#^ *p* < 0.05, ^##^ *p* < 0.01, ^###^ *p* < 0.001 vs. the WT mice.

When encountered with oxidative stressors, cells boost their antioxidant capacity to resist increased ROS production and govern cellular redox status. The Nrf2/ARE signaling pathway is one of the most vital transcription mechanisms to keep the balance of redox in cells through upregulating antioxidant genes. As shown in Figure 4A, PM_2.5_ exposure promoted Nrf2 expression in WT mice (*p* < 0.05).

Activities of anti-oxidative enzymes were determined by colorimetric method. As shown in Figure 4, PM_2.5_ exposure for 12 weeks enhanced the expressions of SOD and CAT in the liver of WT mice (*p* < 0.05) (Figure 4B,C). GSH is especially important for organs with intensive exposure to exogenous toxins, such as the liver, playing an essential role in the detoxification of oxygen-derived free radicals [43]. As shown in Figure 4D, no significant difference in hepatic GSH levels was observed between WT-FA group and Nrf2-FA group. However, PM_2.5_ exposure for 12 weeks significantly decreased hepatic GSH levels in Nrf2^−/−^ mice. These results suggested that Nrf2^−/−^ mice are more susceptible to liver injury in response to PM_2.5_-induced oxidative stress.

In addition, HO-1, an antioxidant enzyme mediated by Nrf2, was also measured from protein and mRNA levels. Nrf2 deletion suppressed protein and mRNA expression of HO-1 and the levels of HO-1 between Nrf2-PM_2.5_ group and Nrf2-FA group had no obvious difference (Figure 4E–G). However, PM_2.5_ exposure accelerated the protein and mRNA expression of HO-1 in WT mice (*p* < 0.05) (Figure 4F,G). These results further showed that PM_2.5_ exposure induced oxidative responses and activated Nrf2/ARE signal pathway in the liver of mice. No significant changes of the levels of malondialdehyde (MDA) and 8-hydroxydeoxyguanosine (8-OHdG) were observed (data not shown).

### 3.6. PM_2.5_ Exposure Activated JNK Signaling Pathway in the Liver Mice

**Figure 5:**
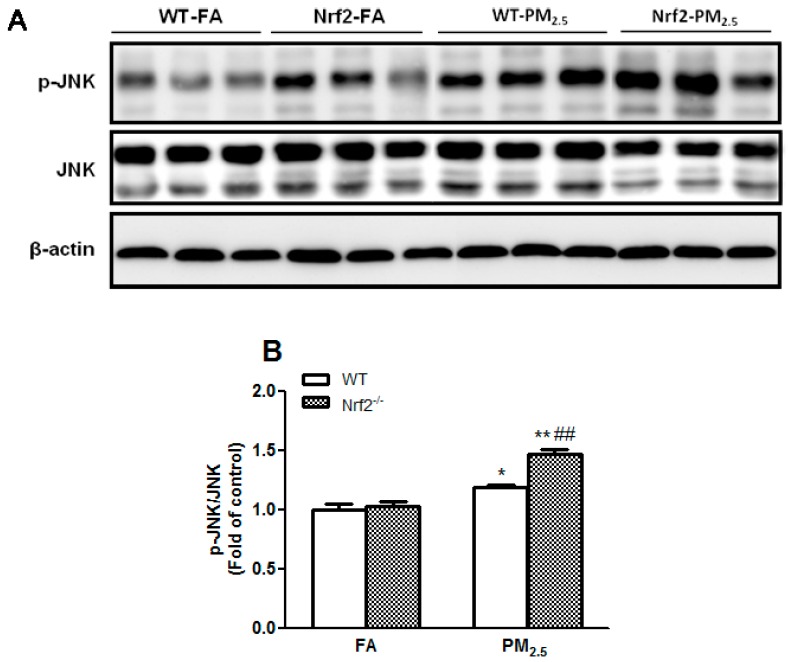
Effects of PM_2.5_ exposure on JNK signal pathway in the liver of WT and Nrf2^−/−^ mice. Animals were exposed to ambient PM_2.5_ or filtered air (FA) for 12 weeks. Representative Western blots (**A**) and quantitative data of phosphor-JNK and JNK (**B**) in liver of mice are shown. β-actin was blotted as a loading control. All values given are the mean ± SE of three independent experiments. * *p* < 0.05, ** *p* < 0.01 vs. the FA groups. ^##^ *p* < 0.01 vs. the WT mice.

JNK is a crucial mediator of insulin resistance, activated by the accumulation of ROS [44,45]. In this study, the phosphorylation of JNK was markedly increased in the liver of mice after PM_2.5_ exposure (*p* < 0.05 and *p* < 0.01) (Figure 5). Meanwhile, Nrf2 deletion further enhanced the phosphorylation level of JNK as compared with the FA groups (*p* < 0.01). The results indicated that PM_2.5_ exposure activated JNK signal pathway in the liver of mice.

### 3.7. PM_2.5_ Exposure Induced Insulin Resistance via Suppressing the IRS-1/AKT Signaling Pathway

**Figure 6:**
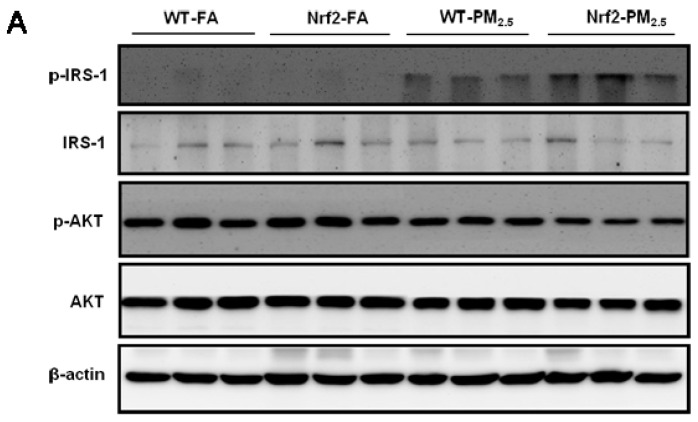

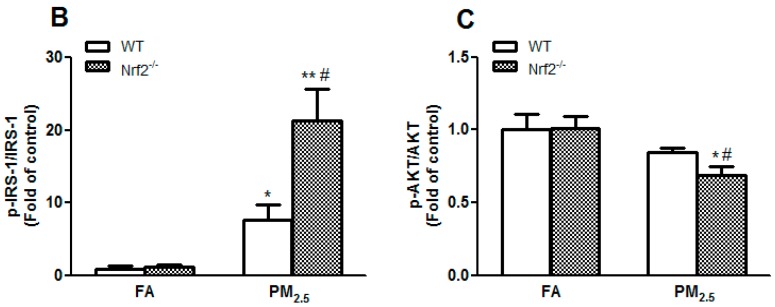
Effects of PM_2.5_ exposure induced insulin resistance-relative IRS-1/AKT phosphorylation in the liver of WT and Nrf2^−/−^ mice. Animals were exposed to ambient PM_2.5_ or filtered air (FA) for 12 weeks. Representative Western blots (**A**) and quantitative data of phosphor-IRS-1, IRS-1, and phosphor-AKT, AKT (**B**,**C**) in liver of mice are shown. β-actin was blotted as a loading control. All values given are the mean ± SE of three independent experiments. * *p* < 0.05, ** *p* < 0.01 vs. the FA groups. ^#^ *p* < 0.05 vs. the WT mice.

JNK activation induces IRS-1 phosphorylation at Ser^307^ and desensitizes insulin action in liver and other tissues, providing a mechanism for JNK mediates feedback inhibition of the insulin signaling cascade [46]. Thus, after we observed that PM_2.5_ exposure increased the phosphorylation of JNK, we then assessed the impacts of PM_2.5_ exposure on IRS-1/AKT signaling pathway. As shown in Figure 6A,B, PM_2.5_ exposure increased the phosphorylation of IRS-1 at Ser^307^ significantly in the liver of mice (*p* < 0.05) and Nrf2 deletion further elevated the level of phosphorylation (*p* < 0.01). In addition, the phosphorylation expression of AKT at Ser^473^ was suppressed significantly in the liver of Nrf2^−/−^ mice exposed to PM_2.5_ for 12 weeks (*p* < 0.05) (Figure 6A,C). These results indicated that PM_2.5_ exposure induced insulin resistance via suppressing IRS-1/AKT signal pathway.

## 4. Discussion

Epidemiological study has shown that increasing diabetes prevalence in the United States is related with increasing PM_2.5_ concentrations [47]. In this study, we found that PM_2.5_ exposure for 12 weeks caused significant liver damage as evidenced by elevated levels of ALT and AST, and induced impaired glucose tolerance, reduced glycogen, and insulin resistance in mice. We further found that PM_2.5_ exposure significantly increased the expressions of Nrf2 and Nrf2-regulated antioxidant genes. Moreover, PM_2.5_ exposure activated the JNK signaling pathway, increased IRS1 phosphorylation at Ser^307^, but reduced AKT phosphorylation at Ser^473^. Taken together, our study demonstrated that PM_2.5_ exposure triggered Nrf2-mediated oxidative responses and activated the JNK-mediated inhibitory signaling pathway, resulting in hepatic insulin resistance.

Oxidative stress has been considered as a causative factor in the development of insulin resistance [34,48]. Many studies have demonstrated that the mechanisms of air pollution-induced health effects involved oxidative stress and inflammation [49,50,51]. A study showed that long-term exposure to ambient fine particulate pollution induced insulin resistance in adipose tissue and decreased glucose tolerance, leading to inflammatory response and oxidative stress, which was evidenced by increasing antioxidant genes regulated by Nrf2 [28]. Recent studies showed that Nrf2 is involved in insulin-mediated glucose uptake, especially under an oxidative status [26]. Activating Nrf2 could intermittently decrease ROS production, enhance insulin sensitivity, and improve insulin resistance [52]. Other study also found that mRNA and protein levels of Nrf2, glutamate-cysteine ligase catalytic subunit (GCLC), a modifier subunit of glutamate cysteine ligase (GCLM), HO-1, and quinone oxidoreductase 1 (NQO-1) were elevated in cerebellum, liver, and lung when the mice were exposed to ambient nanoparticles for a long time, which implicated that ambient particulate matter exposure caused the oxidative stress in organs and tissues, activated the Nrf2 antioxidant signaling pathways [53]. Our previous study has also shown that atmospheric coarse particles could induce human lung epithelial cells A549 producing large amounts of superoxide, hydrogen peroxide, etc., resulting in cellular oxidative stress [54]. In this study, we had not observed significant changes of hepatic levels of MDA and 8-OHdG. However, we did see increased expression of Nrf2 (Figure 4A) and Nrf2-mediated antioxidant enzymes and related peptide, such as SOD (Figure 4B), CAT (Figure 4C), GSH (Figure 4D), and HO-1 (Figure 4G), clearly indicating that PM_2.5_ exposure triggered oxidative responses in the liver.

Glycogen synthase kinase 3 (GSK-3) is a serine/threonine kinase first identified as one of the primary regulators of glycogen synthase (GS) [55]. Elevated GSK-3β activity and expression have been observed in obese and diabetic rodents and humans [56,57]. In this study, a decrease in the contents of hepatic glycogen and the mRNA expressions of GK and GS, and increase in the mRNA expression levels of GSK-3β were observed in the liver of Nrf2^−/−^ mice after PM_2.5_ exposure (Figure 3A–C). It has been demonstrated that the activity of GS is negatively regulated by GSK-3β [58]. GSK-3β controls the switching off of Nrf2 activation of gene expression. GSK3β phosphorylates Fyn, a tyrosine kinase, leading to the nuclear localization of Fyn. Fyn phosphorylates Nrf2 tyrosine 568, resulting in the nuclear export of Nrf2, binding with Keap1, and degradation of Nrf2. The negative regulation of Nrf2 by GSK3β/Fyn is important in repressing Nrf2 downstream genes that were induced in response to oxidative/electrophilic stress [59].

It has been reported that the JNK pathway plays a crucial role in the progression of insulin resistance [60,61]. Activated JNK decreased insulin sensibility via increasing IRS-1 serine^307^ phosphorylation insulin target tissues while insulin resistance status was improved in JNK-KO mice [62]. It has been known the JNK pathway can be activated by several factors, including oxidative stress under diabetes condition [63]. A study showed that PM_2.5_ exposure for 10 weeks increased the levels of phosphorylation of JNK in WT mice [19]. However, in this study they did not give the reasons for how PM_2.5_ activated the JNK signal pathway. In our study, we also found that PM_2.5_ exposure increased the phosphorylation of JNK on Thr^183^/Tyr^185^. Nrf2 deletion further elevated the levels of phosphorylation of JNK, suggesting that PM_2.5_-induced oxidative stress activated the JNK signal pathway. In addition, recent studies have demonstrated that blood inflammatory cytokine TNF-α is involved in hepatic JNK activity [64] and/or insulin resistance [65]. In this study, we found that TNF-α levels in serum were increased during the fourth week, but no significant changes were seen during the 12th week (data not shown). These observations are consistent with the previous reports that acute PM exposure significantly increases inflammatory cytokine levels [66]. There were no significant changes in the levels of inflammatory cytokines after long-term PM_2.5_ exposure [28,67].

It is well known that the IRS-1/AKT signal pathway is a crucial classical insulin signal pathway in the metabolism of glucose [29]. It is generally discussed in studies related to diabetes. In diet-induced obese mice, insulin resistance was induced through the impaired PI3K/AKT signal pathway [33]. However, the detailed mechanisms of PM_2.5_ exposure on insulin resistance remain unclear. In our study, we found that PM_2.5_ exposure increased the phosphorylation expression level of IRS-1 at Ser^307^ significantly and suppressed the phosphorylation of AKT in the livers of Nrf2^−/−^ mice. Meanwhile, there was also an existing decreased tendency for the phosphorylation of AKT in WT mice after PM_2.5_ exposure. These results were consistent with Petra’s study that short-term exposure to PM_2.5_ induces vascular insulin resistance and suppressed insulin-stimulated AKT phosphorylation in mice [68]. These results suggested that the activated JNK pathway after PM_2.5_ exposure inhibited the IRS-1/AKT signal pathway, leading to insulin resistance in the liver of mice.

## 5. Conclusions

In summary, our data showed that PM_2.5_ exposure for 12 weeks caused significant liver damage, and increased the expressions of Nrf2 and Nrf2-regulated antioxidant genes in mice. Moreover, PM_2.5_ exposure activated the JNK-mediated inhibitory signaling pathway, resulting in hepatic insulin resistance. These findings provide insight into how air pollution might increase susceptibility to metabolic diseases, especially type 2 diabetes.

## Figures and Tables

**Table 1:** Primers for Real-time polymerase chain reaction (PCR) analysis.

Genes	Forward Primer (5′-3′)	Reverse Primer (5′-3′)
*Nrf2*	CTGAACTCCTGGACGGGACTA	CGGTGGGTCTCCGTAAATGG
*HO-1*	GATAGAGCGCAACAAGCAGAA	CAGTGAGGCCCATACCAGAAG
*GS*	ACCAAGGCCAAAACGACAG	GGGCTCACATTGTTCTACTTGA
*GSK-3β*	ACCCTCATTACCTGACCTT	CTCAACTTAACAGACGGCT
*GK*	GGAACCAACTTCAGGGTGATG	CTGGTGTTTCGTCTTCACGCT
*Actin*	GTGACGTTGACATCCGTAAAGA	GCCGGACTCATCGTACTCC

**Table 2:** Effects of PM_2.5_ on body weight, liver weight, fasting glucose, and insulin in mice.

Groups	Final Body Weight (g)	Food Consumption (g/mouse/day)	Water Consumption (g/mouse/day)	Liver Weight (g)	Glucose (mmol/L)	Insulin (μIU/mL)
WT-FA	30.8 ± 0.76	4.27	4.33	1.16 ± 0.03	7.3 ± 0.31	12.7 ± 1.60
WT-PM_2.5_	29.9 ± 1.18	4.19	3.59	1.26 ± 0.06 *	7.5 ± 0.59	14.4 ± 1.62
Nrf2-FA	29.3 ± 0.58	5.44	3.82	1.11 ± 0.05	7.5 ± 0.23	13.5 ± 1.14
Nrf2-PM_2.5_	29.9 ± 0.68	4.66	2.87	1.21 ± 0.10	8.0 ± 0.61 *	20.0 ± 0.40 *^,#^

Data are mean ± SEM (*n* = 6). * *p* < 0.05 vs. the FA group; ^#^ *p* < 0.05 vs. the WT mice. FA means filtered air.
